# Supplementary material for: Severe cerebellar malformations in mutant mice demonstrate a role for PDGF-C/PDGFRα signalling in cerebellar development
Source: Biol Open. 2022 Aug 9;11(8):bio059431. doi: 10.1242/bio.059431 (PMC9382116; doi:10.1242/bio.059431)
Supplement: Supplementary information [file biolopen-11-059431-s1.pdf]

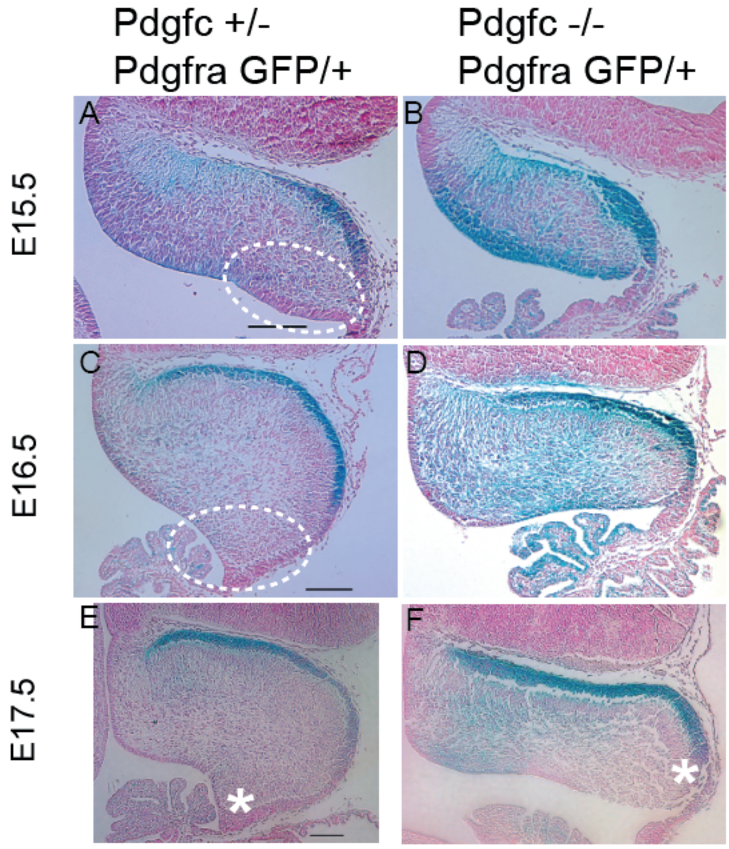

Fig. S1. Lack of expansion in the rhombic lip area in *Pdgfc$^{-/-}$; Pdgfra$^{GFP/+}$* mice. X-gal staining as a reporter for *Pdgfc* expression in mid-sagittal sections of cerebellum of prenatal pups. (A, C, E) In control mice, the area close to the rhombic lip expanded and the cerebellum acquired a rounded shape. *Pdgfc* expression was restricted to granule cells in the EGL. (B, D, F) Cerebellum in mutant mice remained flat and elongated. As a result, the rhombic lip and the choroid plexus was dorsally located (asterisk). Each section is a representative of >3 mice.

Biology Open • Supplementary information

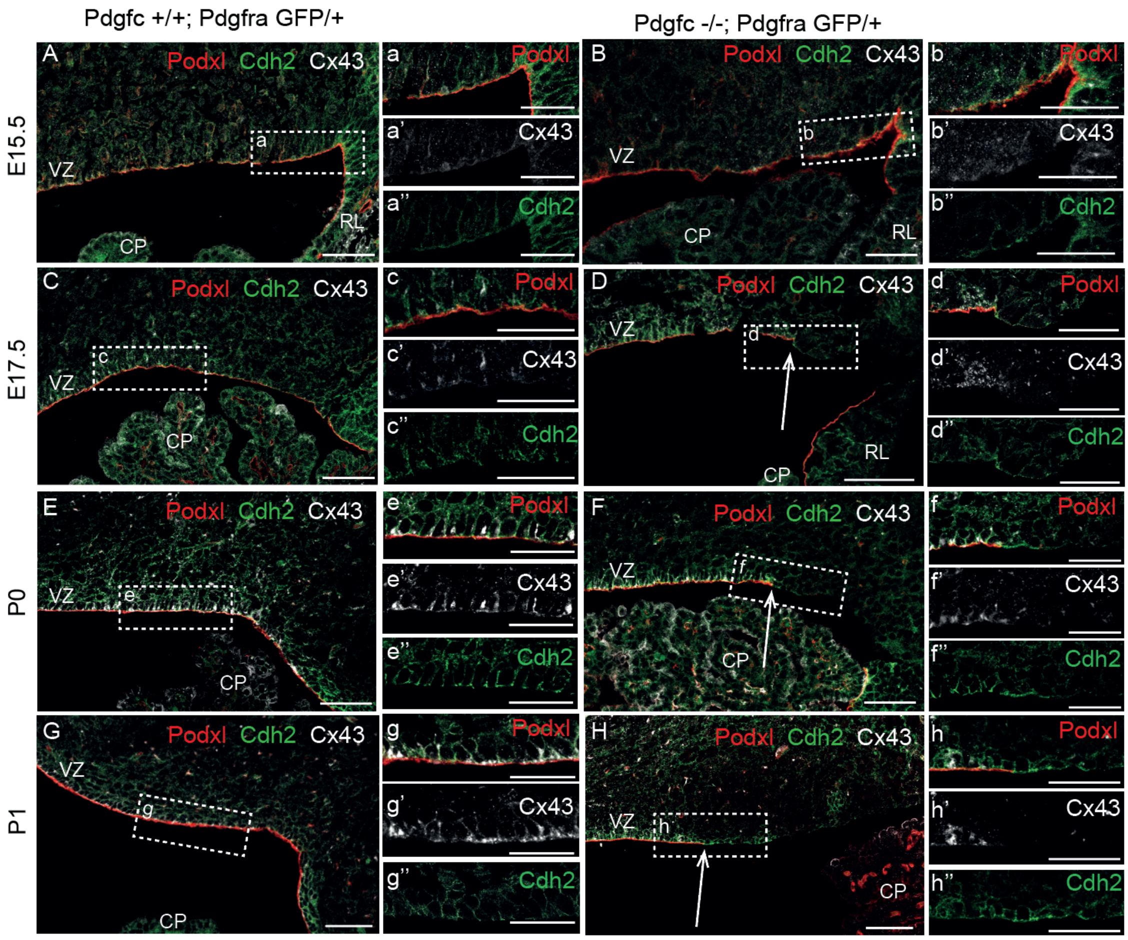

Fig. S2. Alterations in expression of ependyma-specific markers Immunofluorescent staining of Cdh2 and Cx43 in mid-sagittal sections of the ventricular zone at E15.5, E17.5, P0 and P1. (A, C, E, G) In control mice, podocalyxin was expressed in the apical side of ependymal cells. Cdh2 and Cx43 expression was expressed paracellular of the ependymal cells. (a, c, e, g) High magnification view. (D, F, H) From E17.5, podocalyxin expression did not cover the whole ventricular zone. Expression of Cx43 was lost and expression of Cdh2 was reduced. VZ-ventricular zone, CP-choroid plexus, RL-rhombic lip. Arrows indicate where podocalyxin expression was lost. Scalebar (A-H) 50 μm, (a-h) 30 μm. Each section is a representative of >3 mice.

Table S1. E14.5 differentially expressed genes

Click here to download Table S1

Table S2. E14.5 downregulated GO terms

GOBPID	Pvalue	OddsRatio	ExpCount	Count	Size	Term
GO:0032501	1,566E-18	1,61	511,28	669	8198	multicellular organismal process
GO:0007186	1,541E-15	1,95	120,37	208	1930	G-protein coupled receptor signaling pathway
GO:0003008	2,043E-15	1,76	188,85	293	3028	system process
GO:0007608	3,341E-15	2,23	71,28	141	1143	sensory perception of smell
GO:0007600	7,041E-14	1,87	123,36	206	1978	sensory perception
GO:0007165	5,694E-13	1,52	380,43	500	6100	signal transduction
GO:0050896	1,863E-12	1,46	584,62	713	9374	response to stimulus
GO:0050877	3,617E-11	1,68	149,80	228	2402	nervous system process
GO:0007154	1,051E-10	1,44	421,35	531	6756	cell communication
GO:0023052	1,993E-10	1,43	417,36	525	6692	signaling
GO:0065007	3,144E-10	1,40	769,54	885	12339	biological regulation
GO:0007606	5,181E-10	1,81	90,68	150	1454	sensory perception of chemical stimulus
GO:0006954	6,064E-10	2,25	40,91	83	656	inflammatory response
GO:0032101	3,098E-09	2,13	44,47	86	713	regulation of response to external stimulus
GO:0051716	7,644E-09	1,37	484,09	585	7762	cellular response to stimulus
GO:0006952	8,761E-09	1,70	100,16	157	1606	defense response
GO:0050789	1,252E-08	1,35	732,55	837	11746	regulation of biological process
GO:0006816	2,736E-08	2,46	24,88	55	399	calcium ion transport
GO:0043269	3,033E-08	2,08	41,60	79	667	regulation of ion transport
GO:0050727	6,01E-08	2,63	19,58	46	314	regulation of inflammatory response
GO:0051480	8,289E-08	2,54	21,08	48	338	regulation of cytosolic calcium ion concentration
GO:0001819	1,03E-07	2,37	25,20	54	404	positive regulation of cytokine production
GO:0006812	1,115E-07	1,80	65,61	109	1052	cation transport
GO:0032103	1,712E-07	2,66	17,71	42	284	positive regulation of response to external stimulus
GO:0070838	1,981E-07	2,26	27,75	57	445	divalent metal ion transport
GO:0072511	2,482E-07	2,24	27,94	57	448	divalent inorganic cation transport
GO:0051606	3,256E-07	2,18	29,56	59	474	detection of stimulus
GO:0007204	3,586E-07	2,54	18,83	43	302	positive regulation of cytosolic calcium ion concentration
GO:0001817	3,703E-07	1,99	39,73	73	637	regulation of cytokine production
GO:0001816	3,89E-07	1,93	44,22	79	709	cytokine production
GO:0009605	5,036E-07	1,50	145,69	203	2336	response to external stimulus
GO:0032760	6,243E-07	4,51	5,18	19	83	positive regulation of tumor necrosis factor production
GO:1903557	7,6E-07	4,44	5,24	19	84	positive regulation of tumor necrosis factor superfamily cytokine production
GO:0032680	8,603E-07	3,46	8,42	25	135	regulation of tumor necrosis factor production
GO:0031347	9,697E-07	2,02	34,86	65	559	regulation of defense response
GO:0032640	1,143E-06	3,40	8,54	25	137	tumor necrosis factor production
GO:1903555	1,143E-06	3,40	8,54	25	137	regulation of tumor necrosis factor superfamily cytokine production
GO:0006811	1,273E-06	1,59	93,36	139	1497	ion transport
GO:0030001	1,534E-06	1,81	51,14	86	820	metal ion transport
GO:0071706	1,729E-06	3,31	8,73	25	140	tumor necrosis factor superfamily cytokine production
GO:0006874	2,123E-06	2,14	27,00	53	433	cellular calcium ion homeostasis
GO:0034765	2,221E-06	2,12	27,75	54	445	regulation of ion transmembrane transport
GO:0098660	2,581E-06	1,88	41,85	73	671	inorganic ion transmembrane transport
GO:0055074	2,721E-06	2,10	27,94	54	448	calcium ion homeostasis
GO:0080134	3,103E-06	1,64	74,34	114	1192	regulation of response to stress
GO:0030183	3,801E-06	3,14	9,11	25	146	B cell differentiation
GO:0002367	4,042E-06	4,08	5,30	18	85	cytokine production involved in immune response
GO:0072503	5,597E-06	2,04	28,63	54	459	cellular divalent inorganic cation homeostasis
GO:0050794	7,273E-06	1,26	692,64	774	11106	regulation of cellular process
GO:0002532	8,398E-06	4,55	4,05	15	65	production of molecular mediator involved in inflammatory response
GO:0002718	8,398E-06	4,55	4,05	15	65	regulation of cytokine production involved in immune response
GO:0051924	8,895E-06	2,44	15,84	35	254	regulation of calcium ion transport
GO:0051047	1,015E-05	1,97	30,68	56	492	positive regulation of secretion
GO:0030003	1,028E-05	1,86	38,17	66	612	cellular cation homeostasis

Biology Open • Supplementary information

GO:0072507	1,129E-05	1,97	30,06	55	482	divalent inorganic cation homeostasis
GO:0032722	1,185E-05	4,71	3,68	14	59	positive regulation of chemokine production
GO:0006873	1,192E-05	1,84	39,10	67	627	cellular ion homeostasis
GO:0050729	1,201E-05	3,29	7,36	21	118	positive regulation of inflammatory response
GO:0070588	1,287E-05	2,36	16,78	36	269	calcium ion transmembrane transport
GO:0034220	1,323E-05	1,69	55,63	88	892	ion transmembrane transport
GO:0098662	1,498E-05	1,84	37,86	65	607	inorganic cation transmembrane transport
GO:0009593	1,516E-05	2,17	21,02	42	337	detection of chemical stimulus
GO:0050878	1,516E-05	2,17	21,02	42	337	regulation of body fluid levels
GO:0097530	1,518E-05	3,12	8,05	22	129	granulocyte migration
GO:0046651	1,805E-05	2,23	19,08	39	306	lymphocyte proliferation
GO:0050728	1,839E-05	2,99	8,73	23	140	negative regulation of inflammatory response
GO:0032943	2,097E-05	2,21	19,21	39	308	mononuclear cell proliferation
GO:1901741	2,196E-05	9,30	1,31	8	21	positive regulation of myoblast fusion
GO:0060143	2,338E-05	7,56	1,68	9	27	positive regulation of syncytium formation by plasma membrane fusion
GO:0051239	2,344E-05	1,36	186,54	239	2991	regulation of multicellular organismal process
GO:0055080	2,396E-05	1,75	43,84	72	703	cation homeostasis
GO:0002697	2,597E-05	2,10	22,20	43	356	regulation of immune effector process
GO:0002376	2,721E-05	1,38	161,90	211	2596	immune system process
GO:0098771	2,814E-05	1,74	44,84	73	719	inorganic ion homeostasis
GO:1900015	2,844E-05	6,30	2,12	10	34	regulation of cytokine production involved in inflammatory response
GO:0002685	2,984E-05	2,61	11,48	27	184	regulation of leukocyte migration
GO:0015696	3,098E-05	3,16	7,23	20	116	ammonium transport
GO:0098655	3,174E-05	1,75	42,66	70	684	cation transmembrane transport
GO:0046209	3,678E-05	3,92	4,55	15	73	nitric oxide metabolic process
GO:0010959	3,934E-05	2,00	24,76	46	397	regulation of metal ion transport
GO:0055085	4,082E-05	1,53	78,77	114	1263	transmembrane transport
GO:0050801	4,083E-05	1,69	48,52	77	778	ion homeostasis
GO:0034762	4,106E-05	1,84	33,74	58	541	regulation of transmembrane transport
GO:0045321	4,325E-05	1,62	59,06	90	947	leukocyte activation
GO:0002252	4,379E-05	1,67	51,02	80	818	immune effector process
GO:0002687	4,454E-05	2,88	8,61	22	138	positive regulation of leukocyte migration
GO:0006875	4,798E-05	1,83	33,93	58	544	cellular metal ion homeostasis
GO:0032757	4,812E-05	5,20	2,68	11	43	positive regulation of interleukin-8 production
GO:0098542	4,832E-05	1,74	41,66	68	668	defense response to other organism
GO:0051049	4,835E-05	1,43	118,43	160	1899	regulation of transport
GO:0001775	4,85E-05	1,58	65,80	98	1055	cell activation
GO:0010831	4,917E-05	5,81	2,25	10	36	positive regulation of myotube differentiation
GO:0097529	4,957E-05	2,58	11,16	26	179	myeloid leukocyte migration
GO:0042110	4,968E-05	1,88	30,19	53	484	T cell activation
GO:0051046	5,195E-05	1,65	52,08	81	835	regulation of secretion
GO:0070661	5,312E-05	2,11	20,02	39	321	leukocyte proliferation
GO:0050900	5,433E-05	2,13	19,33	38	310	leukocyte migration
GO:1901019	5,45E-05	3,57	5,24	16	84	regulation of calcium ion transmembrane transporter activity
GO:0045087	6,149E-05	1,63	54,76	84	878	innate immune response
GO:0031349	6,243E-05	2,11	19,46	38	312	positive regulation of defense response
GO:0002534	6,363E-05	5,60	2,31	10	37	cytokine production involved in inflammatory response
GO:1901739	6,706E-05	7,55	1,50	8	24	regulation of myoblast fusion
GO:0044703	7,109E-05	1,60	59,06	89	947	multi-organism reproductive process
GO:0071608	7,161E-05	60,30	0,31	4	5	macrophage inflammatory protein-1 alpha production
GO:0140052	7,161E-05	60,30	0,31	4	5	cellular response to oxidised low-density lipoprotein particle stimulus
GO:0046903	7,207E-05	1,54	71,47	104	1146	secretion
GO:0019233	7,588E-05	2,84	8,29	21	133	sensory perception of pain
GO:0050953	7,718E-05	2,76	8,92	22	143	sensory perception of light stimulus
GO:0055065	8,085E-05	1,73	39,23	64	629	metal ion homeostasis
GO:0051607	8,123E-05	2,36	13,47	29	216	defense response to virus
GO:2001057	8,237E-05	3,61	4,86	15	78	reactive nitrogen species metabolic process
GO:0002682	8,489E-05	1,49	85,25	120	1367	regulation of immune system process

GO:0002699	8,612E-05	2,39	12,85	28	206	positive regulation of immune effector process
GO:0006809	9,043E-05	3,78	4,37	14	70	nitric oxide biosynthetic process
GO:0072677	9,324E-05	7,11	1,56	8	25	eosinophil migration
GO:0007193	9,588E-05	3,55	4,93	15	79	adenylate cyclase-inhibiting G-protein coupled receptor signaling pathway
GO:0042100	9,931E-05	2,97	7,23	19	116	B cell proliferation
GO:0051704	9,986E-05	1,38	136,89	179	2195	multi-organism process
GO:0002700	0,0001034	2,86	7,86	20	126	regulation of production of molecular mediator of immune response
GO:0032733	0,0001054	5,91	2,00	9	32	positive regulation of interleukin-10 production
GO:0060142	0,0001054	5,91	2,00	9	32	regulation of syncytium formation by plasma membrane fusion
GO:0006955	0,0001114	1,44	99,54	136	1596	immune response
GO:0002684	0,0001128	1,56	61,49	91	986	positive regulation of immune system process
GO:0042108	0,0001144	3,93	3,93	13	63	positive regulation of cytokine biosynthetic process
GO:0042136	0,0001252	3,14	6,17	17	99	neurotransmitter biosynthetic process
GO:0002444	0,0001287	3,28	5,61	16	90	myeloid leukocyte mediated immunity
GO:0006950	0,000131	1,30	223,58	274	3585	response to stress
GO:0032637	0,0001355	3,86	3,99	13	64	interleukin-8 production
GO:0044130	0,0001364	8,13	1,25	7	20	negative regulation of growth of symbiont in host
GO:0032653	0,0001418	4,50	2,99	11	48	regulation of interleukin-10 production
GO:1903169	0,0001433	2,63	9,29	22	149	regulation of calcium ion transmembrane transport
GO:0007601	0,0001447	2,70	8,67	21	139	visual perception
GO:0032642	0,0001487	3,39	5,11	15	82	regulation of chemokine production
GO:0002688	0,0001517	2,97	6,86	18	110	regulation of leukocyte chemotaxis
GO:0048878	0,0001581	1,51	69,60	100	1116	chemical homeostasis
GO:0030098	0,0001685	1,91	24,07	43	386	lymphocyte differentiation
GO:0048871	0,0001738	1,96	21,89	40	351	multicellular organismal homeostasis
GO:0042133	0,0001776	2,65	8,79	21	141	neurotransmitter metabolic process
GO:0002526	0,0001783	2,73	8,17	20	131	acute inflammatory response
GO:0044126	0,0001936	7,55	1,31	7	21	regulation of growth of symbiont in host
GO:0046649	0,0001969	1,60	50,95	77	817	lymphocyte activation
GO:0061041	0,0001981	2,71	8,23	20	132	regulation of wound healing
GO:0050906	0,0001991	1,91	23,51	42	377	detection of stimulus involved in sensory perception
GO:0006953	0,0002021	4,72	2,62	10	42	acute-phase response
GO:0009410	0,0002113	2,25	13,53	28	217	response to xenobiotic stimulus
GO:0002702	0,0002251	3,24	5,30	15	85	positive regulation of production of molecular mediator of immune response
GO:1903409	0,000232	2,96	6,49	17	104	reactive oxygen species biosynthetic process
GO:0008283	0,0002372	1,37	124,11	162	1990	cell proliferation
GO:0032677	0,0002398	3,86	3,68	12	59	regulation of interleukin-8 production
GO:1903034	0,0002448	2,46	10,29	23	165	regulation of response to wounding
GO:0050701	0,0002487	4,58	2,68	10	43	interleukin-1 secretion
GO:0032613	0,0002515	4,16	3,18	11	51	interleukin-10 production
GO:0032602	0,0002571	3,20	5,36	15	86	chemokine production
GO:0032102	0,0002648	1,99	19,40	36	311	negative regulation of response to external stimulus
GO:0044146	0,0002687	7,04	1,37	7	22	negative regulation of growth of symbiont involved in interaction with host
GO:0010566	0,0002708	9,05	1,00	6	16	regulation of ketone biosynthetic process
GO:0002521	0,0002717	1,71	34,61	56	555	leukocyte differentiation
GO:0051240	0,0002763	1,39	108,64	144	1742	positive regulation of multicellular organismal process
GO:0042127	0,0002777	1,40	102,53	137	1644	regulation of cell proliferation
GO:0002274	0,0002833	2,33	11,72	25	188	myeloid leukocyte activation
GO:0015850	0,0002868	2,20	13,78	28	221	organic hydroxy compound transport
GO:0002690	0,000293	3,15	5,43	15	87	positive regulation of leukocyte chemotaxis
GO:0050810	0,0002982	3,51	4,30	13	69	regulation of steroid biosynthetic process
GO:0051155	0,0002982	3,51	4,30	13	69	positive regulation of striated muscle cell differentiation
GO:0050777	0,0003068	2,47	9,79	22	157	negative regulation of immune response
GO:0002703	0,0003105	2,27	12,47	26	200	regulation of leukocyte mediated immunity
GO:0031348	0,0003105	2,27	12,47	26	200	negative regulation of defense response
GO:0050663	0,0003109	2,23	13,16	27	211	cytokine secretion

GO:0019953	0,0003222	1,58	50,14	75	804	sexual reproduction
GO:0010830	0,0003325	3,70	3,80	12	61	regulation of myotube differentiation
GO:1904062	0,0003388	1,96	19,65	36	315	regulation of cation transmembrane transport
GO:0032612	0,0003444	3,26	4,93	14	79	interleukin-1 production
GO:0002888	0,0003524	4,86	2,31	9	37	positive regulation of myeloid leukocyte mediated immunity
GO:0042738	0,000359	3,96	3,31	11	53	exogenous drug catabolic process
GO:0044144	0,0003655	6,60	1,43	7	23	modulation of growth of symbiont involved in interaction with host
GO:1903530	0,0003803	1,59	47,15	71	756	regulation of secretion by cell
GO:0032879	0,0003889	1,31	169,51	211	2718	regulation of localization
GO:0045428	0,0003893	3,63	3,87	12	62	regulation of nitric oxide biosynthetic process
GO:0002275	0,0003939	3,21	4,99	14	80	myeloid cell activation involved in immune response
GO:0043271	0,0004012	2,42	9,98	22	160	negative regulation of ion transport
GO:1903532	0,0004093	1,78	27,38	46	439	positive regulation of secretion by cell
GO:0006936	0,0004096	2,01	17,65	33	283	muscle contraction
GO:0007200	0,0004106	2,80	6,80	17	109	phospholipase C-activating G-protein coupled receptor signaling pathway
GO:0002250	0,000423	1,71	32,87	53	527	adaptive immune response
GO:0002720	0,0004365	4,69	2,37	9	38	positive regulation of cytokine production involved in immune response
GO:0055082	0,0004414	1,58	47,40	71	760	cellular chemical homeostasis
GO:0032940	0,000468	1,50	61,62	88	988	secretion by cell
GO:0050818	0,0005112	3,12	5,11	14	82	regulation of coagulation
GO:1900225	0,0005126	10,77	0,75	5	12	regulation of NLRP3 inflammasome complex assembly
GO:0071466	0,0005277	2,48	8,86	20	142	cellular response to xenobiotic stimulus
GO:0048583	0,0005538	1,26	228,63	274	3666	regulation of response to stimulus
GO:0009582	0,0005611	2,62	7,61	18	122	detection of abiotic stimulus
GO:2001023	0,0005623	2,82	6,36	16	102	regulation of response to drug
GO:0019221	0,0005702	1,83	23,20	40	372	cytokine-mediated signaling pathway
GO:0048584	0,0005779	1,34	131,90	168	2115	positive regulation of response to stimulus
GO:0032755	0,0005802	3,07	5,18	14	83	positive regulation of interleukin-6 production
GO:0050715	0,000602	2,53	8,29	19	133	positive regulation of cytokine secretion
GO:0019373	0,0006134	5,03	2,00	8	32	epoxygenase P450 pathway
GO:0036230	0,0006134	5,03	2,00	8	32	granulocyte activation
GO:0009581	0,0006197	2,60	7,67	18	123	detection of external stimulus
GO:0032649	0,0006278	2,78	6,42	16	103	regulation of interferon-gamma production
GO:0044117	0,0006421	5,87	1,56	7	25	growth of symbiont in host
GO:0003012	0,0006677	1,82	23,39	40	375	muscle system process
GO:0002366	0,0006749	2,02	15,97	30	256	leukocyte activation involved in immune response
GO:0032409	0,0006749	2,02	15,97	30	256	regulation of transporter activity
GO:0015844	0,0006875	2,87	5,86	15	94	monoamine transport
GO:0061061	0,0007114	1,59	41,66	63	668	muscle structure development
GO:0032490	0,0007903	9,42	0,81	5	13	detection of molecule of bacterial origin
GO:0035815	0,0007903	9,42	0,81	5	13	positive regulation of renal sodium excretion
GO:0042535	0,0007903	9,42	0,81	5	13	positive regulation of tumor necrosis factor biosynthetic process
GO:0044546	0,0007903	9,42	0,81	5	13	NLRP3 inflammasome complex assembly
GO:0002886	0,0008055	3,54	3,62	11	58	regulation of myeloid leukocyte mediated immunity
GO:0042107	0,0008526	2,60	7,23	17	116	cytokine metabolic process
GO:0008015	0,0008564	1,70	29,13	47	467	blood circulation
GO:0045348	0,0008607	15,07	0,50	4	8	positive regulation of MHC class II biosynthetic process
GO:0019218	0,0008612	2,80	5,99	15	96	regulation of steroid metabolic process
GO:1990266	0,000865	2,69	6,61	16	106	neutrophil migration
GO:0002263	0,0008695	1,98	16,22	30	260	cell activation involved in immune response
GO:0032412	0,0008824	2,03	14,78	28	237	regulation of ion transmembrane transporter activity
GO:0050907	0,0009036	1,89	19,21	34	308	detection of chemical stimulus involved in sensory perception
GO:0043315	0,0009233	45,19	0,25	3	4	positive regulation of neutrophil degranulation
GO:0071640	0,0009233	45,19	0,25	3	4	regulation of macrophage inflammatory protein 1 alpha production
GO:0071724	0,0009233	45,19	0,25	3	4	response to diacyl bacterial lipopeptide

GO:0071726	0,0009233	45,19	0,25	3	4	cellular response to diacyl bacterial lipopeptide
GO:1902565	0,0009233	45,19	0,25	3	4	positive regulation of neutrophil activation
GO:0009615	0,0009252	1,97	16,28	30	261	response to virus
GO:0061045	0,0009291	3,24	4,24	12	68	negative regulation of wound healing
GO:0051707	0,0009545	1,45	65,61	91	1052	response to other organism
GO:0030595	0,0009809	2,15	12,04	24	193	leukocyte chemotaxis

Table S3. P0 differentially expressed genes

Click here to download Table S3

Table S4. P0 downregulated GO terms

GOBPID	Pvalue	OddsRatio	ExpCount	Count	Size	Term
GO:0072359	3,661E-12	6,24	5,45	27	1082	circulatory system development
GO:0001568	4,771E-12	7,72	3,48	22	692	blood vessel development
GO:0001944	1,062E-11	7,39	3,63	22	721	vasculature development
GO:0072358	1,501E-11	7,26	3,69	22	734	cardiovascular system development
GO:0048514	1,847E-11	8,05	2,99	20	595	blood vessel morphogenesis
GO:0048731	3,878E-11	3,56	23,23	55	4615	system development
GO:0001525	5,284E-11	8,66	2,48	18	492	angiogenesis
GO:0002040	1,134E-10	23,10	0,52	10	103	sprouting angiogenesis
GO:0035295	1,349E-10	5,64	5,45	25	1082	tube development
GO:0035239	3,834E-10	6,06	4,38	22	870	tube morphogenesis
GO:0007275	4,424E-10	3,26	26,25	57	5215	multicellular organism development
GO:0001570	1,031E-09	22,82	0,47	9	93	vasculogenesis
GO:0042127	1,957E-09	4,36	8,27	29	1644	regulation of cell proliferation
GO:0008283	2,273E-09	4,04	10,02	32	1990	cell proliferation
GO:0032502	2,376E-09	3,04	30,63	61	6086	developmental process
GO:0048646	6,155E-09	4,96	5,57	23	1106	anatomical structure formation involved in morphogenesis
GO:0048513	7,032E-09	3,31	16,92	42	3362	animal organ development
GO:0048856	1,146E-08	2,92	28,52	57	5666	anatomical structure development
GO:1903670	2,681E-08	26,66	0,31	7	62	regulation of sprouting angiogenesis
GO:0051239	3,219E-08	3,26	15,05	38	2991	regulation of multicellular organismal process
GO:0007155	4,014E-08	4,45	6,16	23	1224	cell adhesion
GO:0048585	4,789E-08	4,01	7,83	26	1556	negative regulation of response to stimulus
GO:0022610	4,795E-08	4,40	6,22	23	1236	biological adhesion
GO:0009653	5,305E-08	3,32	13,34	35	2650	anatomical structure morphogenesis
GO:1901342	7,011E-08	8,56	1,59	12	316	regulation of vasculature development
GO:0009888	9,422E-08	3,68	9,26	28	1840	tissue development
GO:0050793	1,021E-07	3,27	13,04	34	2590	regulation of developmental process
GO:0001936	1,5E-07	15,33	0,59	8	118	regulation of endothelial cell proliferation
GO:0010648	1,545E-07	4,10	6,64	23	1319	negative regulation of cell communication
GO:0023057	1,63E-07	4,09	6,66	23	1323	negative regulation of signaling
GO:0016477	2,185E-07	4,02	6,77	23	1345	cell migration
GO:0045765	2,559E-07	8,48	1,46	11	290	regulation of angiogenesis
GO:0001935	3,561E-07	13,59	0,66	8	132	endothelial cell proliferation
GO:0043542	3,818E-07	10,97	0,92	9	183	endothelial cell migration
GO:0051240	4,363E-07	3,55	8,77	26	1742	positive regulation of multicellular organismal process
GO:0048523	4,682E-07	2,68	23,38	47	4646	negative regulation of cellular process
GO:0001667	4,801E-07	7,09	1,90	12	378	ameboidal-type cell migration
GO:1904018	5,473E-07	10,49	0,96	9	191	positive regulation of vasculature development
GO:0030334	7,205E-07	4,74	4,11	17	816	regulation of cell migration
GO:0010594	7,293E-07	12,30	0,73	8	145	regulation of endothelial cell migration
GO:0048583	7,683E-07	2,77	18,45	40	3666	regulation of response to stimulus
GO:0010631	8,397E-07	8,50	1,31	10	261	epithelial cell migration
GO:0090132	8,999E-07	8,43	1,32	10	263	epithelium migration
GO:0090130	9,639E-07	8,37	1,33	10	265	tissue migration
GO:0010632	1,161E-06	9,54	1,05	9	209	regulation of epithelial cell migration
GO:0048870	1,196E-06	3,62	7,46	23	1483	cell motility
GO:0051674	1,196E-06	3,62	7,46	23	1483	localization of cell
GO:0050678	1,228E-06	7,18	1,71	11	340	regulation of epithelial cell proliferation
GO:0051179	1,433E-06	2,48	29,16	53	5794	localization
GO:0010646	1,444E-06	2,81	15,99	36	3178	regulation of cell communication
GO:2000145	1,477E-06	4,48	4,33	17	860	regulation of cell motility
GO:0030154	1,637E-06	2,63	20,50	42	4073	cell differentiation
GO:0023051	1,714E-06	2,78	16,11	36	3201	regulation of signaling
GO:2000026	2,386E-06	3,14	10,23	27	2032	regulation of multicellular organismal development
GO:0045766	2,872E-06	10,14	0,88	8	174	positive regulation of angiogenesis
GO:0032101	3,049E-06	4,71	3,59	15	713	regulation of response to external stimulus

GO:0048010	3,089E-06	25,74	0,23	5	45	vascular endothelial growth factor receptor signaling pathway
GO:0040011	3,491E-06	3,29	8,56	24	1700	locomotion
GO:0045785	3,498E-06	6,40	1,91	11	379	positive regulation of cell adhesion
GO:0031589	3,5E-06	7,20	1,54	10	306	cell-substrate adhesion
GO:0022603	4,164E-06	3,95	5,20	18	1033	regulation of anatomical structure morphogenesis
GO:0040012	4,53E-06	4,10	4,71	17	935	regulation of locomotion
GO:0002042	4,744E-06	23,40	0,25	5	49	cell migration involved in sprouting angiogenesis
GO:0051270	4,93E-06	4,07	4,74	17	941	regulation of cellular component movement
GO:0007160	5,297E-06	9,29	0,95	8	189	cell-matrix adhesion
GO:0090287	5,92E-06	7,74	1,28	9	255	regulation of cellular response to growth factor stimulus
GO:0050673	6,268E-06	6,01	2,03	11	403	epithelial cell proliferation
GO:0032501	6,643E-06	2,27	41,26	65	8198	multicellular organismal process
GO:0048869	6,974E-06	2,46	21,66	42	4304	cellular developmental process
GO:0009966	6,981E-06	2,76	13,53	31	2689	regulation of signal transduction
GO:0009968	7,15E-06	3,64	5,96	19	1184	negative regulation of signal transduction
GO:0030335	7,624E-06	5,36	2,49	12	494	positive regulation of cell migration
GO:0050920	8,627E-06	8,66	1,02	8	202	regulation of chemotaxis
GO:0007507	9,072E-06	4,87	2,97	13	591	heart development
GO:0048519	9,217E-06	2,35	25,90	47	5147	negative regulation of biological process
GO:2000147	1,091E-05	5,16	2,58	12	512	positive regulation of cell motility
GO:0051094	1,131E-05	3,30	7,32	21	1455	positive regulation of developmental process
GO:0043410	1,134E-05	5,14	2,59	12	514	positive regulation of MAPK cascade
GO:0030947	1,315E-05	32,69	0,15	4	29	regulation of vascular endothelial growth factor receptor signaling pathway
GO:0051272	1,427E-05	5,02	2,65	12	526	positive regulation of cellular component movement
GO:0016525	1,622E-05	12,43	0,53	6	106	negative regulation of angiogenesis
GO:0043534	1,622E-05	12,43	0,53	6	106	blood vessel endothelial cell migration
GO:0030155	1,781E-05	4,55	3,17	13	630	regulation of cell adhesion
GO:2000181	1,902E-05	12,07	0,55	6	109	negative regulation of blood vessel morphogenesis
GO:0040017	1,953E-05	4,85	2,73	12	543	positive regulation of locomotion
GO:1903587	1,992E-05	76,01	0,06	3	11	regulation of blood vessel endothelial cell proliferation involved in sprouting angiogenesis
GO:0007169	2,215E-05	4,79	2,77	12	550	transmembrane receptor protein tyrosine kinase signaling pathway
GO:0034308	2,23E-05	28,17	0,17	4	33	primary alcohol metabolic process
GO:1901343	2,709E-05	11,30	0,58	6	116	negative regulation of vasculature development
GO:0018108	2,797E-05	6,31	1,56	9	310	peptidyl-tyrosine phosphorylation
GO:0048518	2,808E-05	2,20	29,50	50	5862	positive regulation of biological process
GO:0098609	2,983E-05	4,32	3,33	13	662	cell-cell adhesion
GO:0018212	3,016E-05	6,25	1,58	9	313	peptidyl-tyrosine modification
GO:0071363	3,416E-05	4,57	2,89	12	575	cellular response to growth factor stimulus
GO:0090049	3,544E-05	24,75	0,19	4	37	regulation of cell migration involved in sprouting angiogenesis
GO:1903672	3,544E-05	24,75	0,19	4	37	positive regulation of sprouting angiogenesis
GO:0048568	3,551E-05	4,94	2,45	11	486	embryonic organ development
GO:0048522	3,79E-05	2,21	26,45	46	5255	positive regulation of cellular process
GO:0051241	3,855E-05	3,31	6,14	18	1219	negative regulation of multicellular organismal process
GO:0010562	4,071E-05	3,42	5,58	17	1109	positive regulation of phosphorus metabolic process
GO:0045937	4,071E-05	3,42	5,58	17	1109	positive regulation of phosphate metabolic process
GO:0070848	4,104E-05	4,48	2,95	12	586	response to growth factor
GO:0050679	4,759E-05	8,06	0,95	7	188	positive regulation of epithelial cell proliferation
GO:0010634	5,141E-05	10,02	0,65	6	130	positive regulation of epithelial cell migration
GO:0033674	5,201E-05	4,73	2,55	11	507	positive regulation of kinase activity
GO:0006928	5,388E-05	2,80	9,43	23	1874	movement of cell or subcellular component
GO:0001938	5,606E-05	13,53	0,41	5	81	positive regulation of endothelial cell proliferation
GO:0035924	5,891E-05	21,49	0,21	4	42	cellular response to vascular endothelial growth factor stimulus
GO:0050921	6,345E-05	9,63	0,68	6	135	positive regulation of chemotaxis
GO:0043535	7,064E-05	12,85	0,43	5	85	regulation of blood vessel endothelial cell migration
GO:0002687	7,169E-05	9,41	0,69	6	138	positive regulation of leukocyte migration

GO:0008284	7,42E-05	3,53	4,72	15	937	positive regulation of cell proliferation
GO:0065007	7,669E-05	2,10	62,10	83	12339	biological regulation
GO:0002043	8,03E-05	43,42	0,09	3	17	blood vessel endothelial cell proliferation involved in sprouting angiogenesis
GO:0010595	8,336E-05	12,38	0,44	5	88	positive regulation of endothelial cell migration
GO:0048608	8,336E-05	4,89	2,23	10	443	reproductive structure development
GO:0061458	8,976E-05	4,84	2,25	10	447	reproductive system development
GO:0042572	9,6E-05	40,53	0,09	3	18	retinol metabolic process
GO:0080134	9,884E-05	3,17	6,00	17	1192	regulation of response to stress
GO:0010596	0,0001001	18,56	0,24	4	48	negative regulation of endothelial cell migration
GO:0007166	0,0001021	2,49	12,61	27	2505	cell surface receptor signaling pathway
GO:0019220	0,000105	2,79	8,53	21	1695	regulation of phosphate metabolic process
GO:0051174	0,0001095	2,78	8,56	21	1700	regulation of phosphorus metabolic process
GO:0072001	0,0001153	5,92	1,46	8	291	renal system development
GO:0006954	0,0001198	3,98	3,30	12	656	inflammatory response
GO:0007167	0,0001241	3,53	4,38	14	870	enzyme linked receptor protein signaling pathway
GO:0001974	0,000127	17,37	0,26	4	51	blood vessel remodeling
GO:0048806	0,0001371	17,01	0,26	4	52	genitalia development
GO:0032270	0,0001407	2,88	7,42	19	1474	positive regulation of cellular protein metabolic process
GO:0048014	0,0001497	201,03	0,02	2	4	Tie signaling pathway
GO:0007154	0,0001501	2,01	34,00	53	6756	cell communication
GO:0010647	0,0001563	2,71	8,78	21	1744	positive regulation of cell communication
GO:0023056	0,0001639	2,70	8,81	21	1750	positive regulation of signaling
GO:0002682	0,0001653	2,93	6,88	18	1367	regulation of immune system process
GO:0051093	0,0001677	3,27	5,08	15	1009	negative regulation of developmental process
GO:0006721	0,0001707	16,01	0,28	4	55	terpenoid metabolic process
GO:0051347	0,0001729	4,10	2,92	11	581	positive regulation of transferase activity
GO:0042327	0,0001788	3,25	5,11	15	1015	positive regulation of phosphorylation
GO:0010604	0,000183	2,30	15,27	30	3034	positive regulation of macromolecule metabolic process
GO:0002376	0,0001849	2,39	13,07	27	2596	immune system process
GO:0050727	0,000194	5,47	1,58	8	314	regulation of inflammatory response
GO:0055085	0,0001974	2,98	6,36	17	1263	transmembrane transport
GO:0009887	0,0002009	3,21	5,16	15	1026	animal organ morphogenesis
GO:1903671	0,0002046	30,39	0,12	3	23	negative regulation of sprouting angiogenesis
GO:0032835	0,0002099	15,11	0,29	4	58	glomerulus development
GO:0055080	0,000227	3,70	3,54	12	703	cation homeostasis
GO:0071621	0,0002286	9,87	0,55	5	109	granulocyte chemotaxis
GO:0003158	0,0002593	9,60	0,56	5	112	endothelium development
GO:0072012	0,0002637	27,62	0,13	3	25	glomerulus vasculature development
GO:0043408	0,0002648	3,64	3,60	12	715	regulation of MAPK cascade
GO:0030003	0,0002701	3,88	3,08	11	612	cellular cation homeostasis
GO:0098771	0,0002786	3,62	3,62	12	719	inorganic ion homeostasis
GO:0001655	0,0002883	5,14	1,68	8	333	urogenital system development
GO:0061437	0,000297	26,42	0,13	3	26	renal system vasculature development
GO:0061440	0,000297	26,42	0,13	3	26	kidney vasculature development
GO:0008277	0,0003051	9,25	0,58	5	116	regulation of G-protein coupled receptor protein signaling pathway
GO:0048771	0,0003146	7,08	0,91	6	181	tissue remodeling
GO:0051247	0,0003296	2,68	7,93	19	1576	positive regulation of protein metabolic process
GO:0006873	0,0003317	3,79	3,16	11	627	cellular ion homeostasis
GO:0038084	0,0003329	25,32	0,14	3	27	vascular endothelial growth factor signaling pathway
GO:0050926	0,0003329	25,32	0,14	3	27	regulation of positive chemotaxis
GO:0065008	0,0003379	2,12	18,92	34	3760	regulation of biological quality
GO:0010817	0,0003381	4,08	2,65	10	527	regulation of hormone levels
GO:0002685	0,0003434	6,96	0,93	6	184	regulation of leukocyte migration
GO:0010633	0,0003662	12,95	0,34	4	67	negative regulation of epithelial cell migration
GO:0042592	0,0003693	2,59	8,67	20	1723	homeostatic process
GO:0001934	0,0003697	3,15	4,87	14	968	positive regulation of protein phosphorylation
GO:0001945	0,0003715	24,31	0,14	3	28	lymph vessel development
GO:0032879	0,0003887	2,27	13,68	27	2718	regulation of localization
GO:0009987	0,0004277	2,14	81,19	98	16132	cellular process

GO:0015893	0,0004427	6,62	0,97	6	193	drug transport
GO:0043537	0,0004571	22,50	0,15	3	30	negative regulation of blood vessel endothelial cell migration
GO:0055082	0,0004582	3,41	3,83	12	760	cellular chemical homeostasis
GO:0043549	0,0004691	3,40	3,84	12	762	regulation of kinase activity
GO:0023052	0,0004747	1,90	33,68	51	6692	signaling
GO:0060326	0,0004833	5,44	1,38	7	274	cell chemotaxis
GO:0048878	0,0004893	2,94	5,62	15	1116	chemical homeostasis
GO:0097530	0,0004972	8,27	0,65	5	129	granulocyte migration
GO:0051173	0,0005135	2,20	14,68	28	2916	positive regulation of nitrogen compound metabolic process
GO:0002604	0,0005189	80,40	0,04	2	7	regulation of dendritic cell antigen processing and presentation
GO:1903589	0,0005189	80,40	0,04	2	7	positive regulation of blood vessel endothelial cell proliferation involved in sprouting angiogenesis
GO:0031347	0,0005357	3,84	2,81	10	559	regulation of defense response
GO:0000165	0,000551	3,34	3,91	12	776	MAPK cascade
GO:0006720	0,000563	11,49	0,38	4	75	isoprenoid metabolic process
GO:0050801	0,0005637	3,33	3,92	12	778	ion homeostasis
GO:0090288	0,0005714	8,01	0,67	5	133	negative regulation of cellular response to growth factor stimulus
GO:0030097	0,0005869	3,14	4,51	13	896	hemopoiesis
GO:0009790	0,0005879	2,88	5,72	15	1136	embryo development
GO:0060429	0,0005933	2,88	5,72	15	1137	epithelium development
GO:0098657	0,0005964	3,31	3,94	12	783	import into cell
GO:0032103	0,0005977	5,24	1,43	7	284	positive regulation of response to external stimulus
GO:0050794	0,0006162	1,85	55,90	74	11106	regulation of cellular process
GO:0023014	0,0006448	3,28	3,98	12	790	signal transduction by protein phosphorylation
GO:0009893	0,0006653	2,11	16,46	30	3270	positive regulation of metabolic process
GO:0009967	0,0006822	2,65	7,08	17	1407	positive regulation of signal transduction
GO:0034103	0,0006853	10,87	0,40	4	79	regulation of tissue remodeling
GO:0002468	0,0006896	67,00	0,04	2	8	dendritic cell antigen processing and presentation
GO:0072006	0,0007209	7,60	0,70	5	140	nephron development
GO:0007165	0,0007281	1,88	30,70	47	6100	signal transduction
GO:0090596	0,0007624	5,02	1,49	7	296	sensory organ morphogenesis
GO:0031401	0,0007677	2,80	5,87	15	1166	positive regulation of protein modification process
GO:0048015	0,0007686	7,49	0,71	5	142	phosphatidylinositol-mediated signaling
GO:0034105	0,0007863	18,41	0,18	3	36	positive regulation of tissue remodeling
GO:0001894	0,0008382	5,84	1,10	6	218	tissue homeostasis
GO:0048017	0,0008444	7,32	0,73	5	145	inositol lipid-mediated signaling
GO:0043406	0,0008583	5,81	1,10	6	219	positive regulation of MAP kinase activity
GO:0001775	0,0008613	2,88	5,31	14	1055	cell activation
GO:0060312	0,0008837	57,42	0,05	2	9	regulation of blood vessel remodeling
GO:2001214	0,0008837	57,42	0,05	2	9	positive regulation of vasculogenesis
GO:0042221	0,0008887	1,96	21,58	36	4287	response to chemical
GO:0043085	0,0009014	2,86	5,33	14	1060	positive regulation of catalytic activity
GO:0002702	0,0009023	10,06	0,43	4	85	positive regulation of production of molecular mediator of immune response
GO:1902533	0,0009397	2,98	4,75	13	943	positive regulation of intracellular signal transduction
GO:0022409	0,0009645	5,67	1,13	6	224	positive regulation of cell-cell adhesion
GO:0048534	0,0009673	2,97	4,76	13	946	hematopoietic or lymphoid organ development
GO:0002690	0,0009843	9,82	0,44	4	87	positive regulation of leukocyte chemotaxis
GO:0032526	0,0009843	9,82	0,44	4	87	response to retinoic acid
GO:0034446	0,0009843	9,82	0,44	4	87	substrate adhesion-dependent cell spreading
GO:0015711	0,0009912	4,23	2,02	8	402	organic anion transport
GO:0031325	0,0009973	2,09	15,31	28	3041	positive regulation of cellular metabolic process
GO:0050900	0,0009979	4,79	1,56	7	310	leukocyte migration

Biology Open • Supplementary information

Table S5. Primary antibodies

Name	Host	Company	LOT nr.	Ref. nr.	Reference	Dilution
**Podocalyxin (Podxl)**	goat	R&D Systems	JPC0112111	AF1556	https://www.rndsystems.com/products/mouse-podocalyxin-antibody_af1556	1:200
**GFAP**	rat	Invitrogen		13-0300	https://www.thermofisher.com/order/genome-database/details/antibody/13-0300.html	1:100
**GFAP**	rabbit	Dako	20019135	Z0334	https://www.agilent.com/en/product/immunohistochemistry/antibodies-controls/primary-antibodies/glial-fibrillary-acidic-protein-(concentrate)-76683	1:200
**S100B**	rabbit	Dako	00071715	ZO311	https://www.agilent.com/en/product/immunohistochemistry/antibodies-controls/primary-antibodies/s100-(dako-omnis)-76198	1:100
**Beta IV Tubulin**	rabbit	Abcam	GR252919-6	ab179509	https://www.abcam.com/beta-iv-tubulin-antibody-epr16776-ab179509.html	1:500
**N-cadherin (Cdh2)**	rat	Developmental Studies Hybridoma Bank	6/25/09	MNCD2-C	https://dshb.biology.uiowa.edu/MNCD2	1:50
**Connexin 43 (Cx43)**	mouse	Santa Cruz Biotechnology	I1417	sc-271837	https://www.scbt.com/p/connexin-43-antibody-f-7?gclid=CjwKCAjw0a-SBhBkEiwApljU0jSxW07FG9NXEJSU_GBHQa5HxhamAE4hKZeyVWFvONGm9cfbqFp0PBoCmDsQAvD_BwE	1:50
**Calbindin D-28K**	rabbit	Sigma	093M4801	C2724	https://www.sigmaaldrich.com/SE/en/product/sigma/c2724	1:200

All antibodies were validated in two ways; (1) staining was performed with only secondary antibodies in parallell, (2) staining was verified to appear in cells with correct morphology and in correct anatomical locations.

Biology Open • Supplementary information
